# Relationships of the expression levels of inflammatory factors in serum and cytokines in cerebrospinal fluid with the degree of postoperative nerve injury and prognosis in patients with Glioma

**DOI:** 10.12669/pjms.41.11.12233

**Published:** 2025-11

**Authors:** Dan Zhao, Lei Mi, Yan Chen, Xueshan Sun, Zhanhu Ma, Li Hao

**Affiliations:** 1 Dan Zhao Clinical Laboratory, Baoding No.1 Hospital, Baoding 071000, Hebei, China; 2 Lei Mi Clinical Laboratory, Baoding No.1 Hospital, Baoding 071000, Hebei, China; 3 Yan Chen Department of Neurosurgery, Baoding No.1 Hospital, Baoding 071000, Hebei, China; 4 Xueshan Sun Clinical Laboratory, Baoding No.1 Hospital, Baoding 071000, Hebei, China; 5 Zhanhu Ma Department of Neurosurgery, Baoding No.1 Hospital, Baoding 071000, Hebei, China; 6 Li Hao Clinical Laboratory, Baoding No.1 Hospital, Baoding 071000, Hebei, China

**Keywords:** Cerebrospinal fluid, Cytokines, Inflammatory factors, Nerve injury, Prognosis

## Abstract

**Objective:**

This study aimed to investigate the relationship between serum inflammatory markers (TNF-α, IL-6, CRP) and cerebrospinal fluid (CSF) cytokine levels (AVP, OT, β-EP) with the severity of postoperative neurological injury and prognosis in patients with glioma.

**Methodology:**

In this retrospective study, 80 patients with glioma who underwent surgery (study group) and 80 non-glioma controls who underwent CSF sampling (control group) were enrolled from Baoding No.1 Hospital between May 2023 and December 2024. Serum inflammatory factors (TNF-α, IL-6, CRP) and CSF cytokines (AVP, OT, β-EP) were measured in both groups. Neurological impairment was assessed using the NIH Stroke Scale (NIHSS). Comparative analyses were performed between groups and across different pathological stages and neurological injury severities. Multivariate logistic regression was used to identify risk factors affecting 3-year survival.

**Results:**

The study group showed significantly elevated levels of serum TNF-α, IL-6, CRP and CSF AVP, OT, β-EP compared to controls (p < 0.01). These markers increased with disease severity and higher pathological stage (III–IV vs. I–II, p < 0.01). Multivariate analysis identified TNF-α, IL-6, AVP, β-EP, pathological stage, degree of neurological injury, and extent of resection as independent risk factors for three years survival (p < 0.05).

**Conclusion:**

Elevated levels of serum inflammatory markers and CSF cytokines are associated with more severe neurological injury and worse prognosis in glioma patients. TNF-α, IL-6, AVP, and β-EP are independent risk factors for poor survival outcomes.

## INTRODUCTION

Glioma, a prevalent primary intracranial tumor, poses significant treatment challenges due to its highly invasive nature and poorly defined margins, which often preclude complete surgical resection and contribute to poor prognosis, with an average survival of 9–12 months.^1^-^3^ Despite advances in microsurgical techniques that improve precision and extent of resection, high rates of recurrence and progression persist, underscoring the limitations of current therapeutic strategies.^4^,^5^ Recent evidence highlights the critical role of the tumor microenvironment and neuroinflammatory processes in glioma pathogenesis.^6^ In particular, inflammatory mediators such as TNF-α and IL-6, along with neuropeptides including β-endorphin (β-EP), oxytocin (OT), and arginine vasopressin (AVP), have been implicated in tumor progression and neural damage.^7^,^8^ However, the interplay between systemic inflammation and local neuroendocrine responses in the cerebrospinal fluid (CSF) remains poorly understood, especially in relation to postoperative neurological outcomes and survival. This study aims to elucidate the relationship between serum inflammatory markers, CSF cytokine levels, and postoperative neural injury in glioma patients, with the goal of identifying novel prognostic biomarkers and facilitating more personalized treatment approaches.

## METHODOLOGY

This was a retrospective study. This study was conducted with the inclusion of 80 glioma patients undergoing surgery from May 2023 to December 2024 in Baoding No.1 Hospital (the study group) and 80 subjects without glioma or nerve injury who were subjected to cerebrospinal fluid sampling during the same period (the control group).

### Ethical approval:

The study was approved by the Institutional Ethics Committee of Baoding No.1 Hospital (No.:2023-4-20; date: April 20, 2023), and written informed consent was obtained from all participants.

### Inclusion criteria:

- Patients over 18 years old.
- Patients who met the diagnostic criteria of glioma^9^ and had been confirmed by pathological biopsy.
- Patient with newly diagnosed glioma.
- Patients who did not receive conservative treatment, radiotherapy, chemotherapy or other interventions before surgery.
- Patients with complete clinical and follow-up data.
- Patients without cognitive or mental disorders who were able to understand and actively cooperate with therapeutic schemes.
- Patients who agreed to participate in the study and signed informed consent forms by themselves or their families.

### Exclusion criteria:

- Patients with severe liver and kidney dysfunction.
- Patients with malignant tumors in other parts.
- Patients with chronic medical history of hypertension, diabetes, hyperlipidemia, etc.
- Patients with chronic inflammatory and other diseases that affected the results of study.
- Patients in pregnancy/lactation period.
- Patients with severe mental or cognitive impairments.
- Patients with poor treatment compliance and inability to cooperate with treatment.

The study group including 44 males and 36 females, aged 42-65 years, with an average age of (52.47±5.18) years. In terms of the pathological staging of glioma, there were 14 cases of Stage-I, 35 cases of Stage-II, and 31 cases of stage III; meanwhile, 28 cases had tumors located in the temporal lobe, 30 cases in the parietal lobe, and 22 cases in the frontal lobe. Simultaneously, the control group included 80 participants without glioma or nerve injury who were subjected to cerebrospinal fluid sampling during the same period, including 41 males and 39 females, aged 37-65 years, with an average age of (51.79±4.53) years. There was no significant difference in gender and age between the two groups of patients (p>0.05).

### Laboratory tests:

The fasting venous blood was collected from each subject in our study on the second day after admission and in the morning after surgery. The supernatant was obtained through centrifugation at 3,000r/minutes for 10 minutes. According to the instructions of corresponding testing kits, levels of TNF-α, IL-6, and CRP were detected by enzyme-linked immunosorbent assay, magnetic particle-based chemiluminescence assay, and particle-enhanced turbidimetric immunoassay, respectively. Furthermore, 3ml of cerebrospinal fluid was harvested from each patient through lumbar puncture, followed by centrifugation for 10 minutes after placement at room temperature for one hour. The supernatant was collected and stored at -40°C for future use. Then, the levels of AVP, OT, and β-EP in cerebrospinal fluid of the two groups were detected using radioimmunoassay. All examination results were determined by at least two laboratory physicians to eliminate human error and to ensure their authenticity and accuracy.

### Data Collection of Clinical Outcomes & Survival Rates:

Clinical outcomes, primarily the degree of nerve injury, were assessed using the National Institutes of Health Stroke Scale (NIHSS)^10^ during postoperative hospitalization. Long-term survival data were obtained through a structured three years follow-up protocol. Follow-up was conducted every three months in the first year and every six months in the subsequent two years, via outpatient clinical reviews or telephone interviews. The date of death (if applicable) was confirmed through hospital records or direct communication with family members. According to the prognostic outcome, glioma patients were divided into a death group and a survival group.

### Data verification process:

To ensure accuracy, all survival outcomes and dates were independently verified by two researchers. Any discrepancies were resolved by consulting the original medical records or re-contacting the participant’s family.

### Outcome measures:

- Comparison of the levels of inflammatory factors of TNF-α, IL-6, and CRP, as well as levels of AVP, OT, and β-EP in cerebrospinal fluid between the two groups;
- Comparison of the levels of AVP, OT, and β-EP in cerebrospinal fluid in glioma patients with different degrees of nerve injury;
- Comparison of serum inflammatory factor levels in patients with different postoperative pathological stages;
- Multivariate analysis of prognosis in glioma.

### Statistical analysis:

Data analysis in this study was completed in SPSS 20.0 software (two-tailed a=0.05). The confidence interval was 95%, the measurement data were expressed by *χ̅*±*S*, while the counting data were described as absolute value or composition ratio. Analysis of variance was used for inter-group analysis, and χ^2^ test for rate comparison. The influencing factors were analyzed using logistic regression analysis, and the correlation was analyzed by Pearson correlation analysis. Receiver operating characteristic (ROC) curve was plotted to evaluate the value of various factors in predicting the prognosis of glioma.

## RESULTS

Compared with the control group, the study group had significantly increased levels of TNF-α, IL-6, and CRP in serum, as well as levels of AVP, OT, and β-EP in cerebrospinal fluid, and the differences were statistically significant (p=0.00) (Table-I). According to the correlation (r) analysis of nerve injury with AVP, OT, and β-EP levels, the levels of VP, OT, and β-EP increased with the severity of the disease, showing positive correlations (p<0.01) (Table-II).

**Table-I:** Comparative analysis of inflammatory factors, as well as AVP, OT, and β-EP in cerebrospinal fluid between groups (*χ̅*±*S*, n=80).

Indicators	Study group	Control group	t	p
TNF-α (ng/L)	44.72±11.29	38.70±13.47	3.06	0.00
IL-6 (ng/L)	14.78±5.63	11.03±4.12	4.81	0.00
CRP(mg/L)	76.48±18.95	67.55±11.72	3.58	0.00
AVP (ng/L)	21.76±5.83	18.25±4.75	4.17	0.00
OT (ng/L)	9.38±2.14	7.63±1.50	5.93	0.00
β-EP (ng/L)	94.54±9.42	90.16±9.07	4.25	0.00

p<0.05.

**Table-II:** Correlation (r) analysis of postoperative nerve injury with AVP, OT, and β-EP levels (*χ̅*±*S*, n=80).

Groups	n	AVP (ng/L)	OT (ng/L)	β-EP (ng/L)
Mild	22	0.18	0.22	0.16
Moderate	37	0.23	0.23	0.18
Severe	21	0.27	0.25	0.25
*r*		0.370	0.227	0.322
*p*		<0.01	<0.01	<0.01

The concentrations of inflammatory factors (TNF-α, IL-6, and CRP) were significantly higher in patients with stage III–IV disease compared to those with stage I–II (p < 0.001, Table-III). To identify independent risk factors affecting three-year survival in glioma patients, multiple logistic regression analysis was performed using TNF-α, IL-6, and other relevant indices as independent variables. Variables were assigned as appropriate prior to analysis. The entry and removal criteria for the regression model were set at α = 0.05 and α = 0.10, respectively. The analysis revealed that TNF-α, IL-6, AVP, β-EP, pathological stage, degree of nerve injury, and extent of resection were independent risk factors for three-year survival (all p < 0.05, Table-IV).

**Table-III:** Comparative analysis of serum inflammatory factor levels in patients with different postoperative pathological stages (*χ̅*±*S*, n=80).

Stages	n	TNF-α (ng/L)	CRP (mg/L)	IL-6 (ng/L)
Stage I~II	49	38.94±9.76	72.16±12.20	12.35±4.58
Stage III~IV	31	46.58±10.31	78.67±15.44	15.79±5.73
*t*		4.81	2.96	4.20
*p*		0.00	0.00	0.00

p<0.05.

**Table-IV:** Multivariate Logistic regression analysis of prognosis in glioma (*χ̅*±*S*, n=80).

Variables	β value	SE value	Waldχ^2^	P value	OR value	95.0%CI
TNF-α (ng/L)*	3.507	0.528	4.633	0.000	4.370	3.809~4.824
IL-6 (ng/L)*	4.302	0.430	3.905	0.000	3.289	2.708~3.512
CRP(mg/L)	1.027	0.067	1.273	0.233	0.872	0.407~0.980
AVP (ng/L)*	4.143	0.572	4.405	0.000	3.605	3.219~3.800
OT (ng/L)	0.062	0.014	0.773	0.481	0.841	0.611~0.832
β-EP (ng/L)*	3.785	0.580	3.706	0.000	3.106	2.609~3.348
Age	-0.004	0.031	0.817	0.341	0.543	0.412~0.660
Gender	0.013	0.068	0.582	0.310	0.210	0.125~0.452
Pathological staging*	4.419	0.603	3.802	0.000	3.512	3.210~3.648
Nerve injury degree*	3.289	0.336	3.021	0.000	3.508	3.401~3.784
Total resection*	4.133	0.218	3.509	0.000	3.635	3.604~0.892
Postoperative chemoradiotherapy	0.087	0.223	0.704	0.420	0.355	0.411~0.478

* p<0.05.

## DISCUSSION

Glioma, a highly aggressive primary brain tumor, remains a therapeutic challenge due to its invasive growth and high recurrence rate despite multimodal treatment.^11^,^12^ Intraoperative technologies such as navigation and MRI improve surgical accuracy and resection rates, yet the infiltrative nature of glioma complicates achieving complete resection. Although maximal safe resection is associated with better survival, the optimal resection extent remains uncertain, typically ranging from 95% to 100% for newly diagnosed cases.^13^ This study investigated the levels of CSF neuroendocrine factors (AVP, OT, β-EP) and serum pro-inflammatory cytokines (TNF-α, IL-6, CRP) in glioma patients, and their links to disease severity, nerve injury, and three years survival. Key findings included: significantly higher CSF AVP/OT/β-EP and serum TNF-α/IL-6/CRP in glioma patients than controls (p=0.00); positive correlations between these factors and disease severity (p<0.01); infection-induced further elevation of CSF factors, forming a vicious cycle with nerve injury; and TNF-α, IL-6, AVP, β-EP as independent 3-year survival risk factors (p<0.05).

Consistent with prior international studies, we observed that higher TNF-α, IL-6, and CRP levels correlate with advanced tumor stage and worse outcomes.^14^,^15^ These findings align with the understanding that chronic inflammation promotes immunosuppression and glioma progression. Notably, our results extend current knowledge by establishing a direct relationship between CSF levels of AVP, OT, and β-EP and the degree of neural damage postoperatively—a correlation less commonly documented in existing literature.^16^,^17^ For instance, while Pilozzi et al. highlighted the role of β-EP in neuroinflammation, its specific elevation in CSF following glioma surgery and its association with neurological deficits have been underexplored.^18^ Similarly, although Klemm et al. (2020) described immune alterations in glioma microenvironments, the interplay between systemic inflammation and local neuroendocrine response has not been well quantified.^19^,^20^

A key novel finding of our study is the identification of AVP, β-EP, TNF-α, and IL-6 as independent risk factors for three years survival, suggesting their potential utility as prognostic biomarkers. This adds a new dimension to the conventional prognostic markers based mainly on histology and imaging. The strong correlation between these molecular markers and clinical outcomes may facilitate early intervention in high-risk patients.

### Strengths of the study:

The strengths of our study include a well-characterized patient cohort, standardized sample collection protocols, and the use of multiple analytical methods to ensure data reliability. Nevertheless, several limitations should be acknowledged. The single-center retrospective design may limit generalizability.

### Limitations

The lack of systematic recording and analysis of adverse events represents an important limitation, which may affect the comprehensive safety assessment of postoperative outcomes. In addition, although significant associations were identified, causal relationships cannot be inferred. Future multi-center prospective studies incorporating larger sample sizes, extended molecular profiling, and detailed adverse event records are warranted. Furthermore, research including patients receiving immunotherapy or targeted therapies could clarify whether these biomarkers retain predictive value across treatment modalities.

## CONCLUSIONS

Our findings underscore the critical roles of neuroinflammation and neuroendocrine response in glioma progression and postoperative recovery. The combined assessment of serum inflammatory markers and CSF neuropeptides may offer a novel strategy for prognostic evaluation and personalized treatment planning, ultimately improving clinical outcomes in glioma patients.

### Authors’ Contributions:

**DZ:** Concept and design of study. Literature search.

**LM, YC** and **XS:** Critical revision of manuscript and responsible for data integrity.

**ZM** and **LH:** Data collection, analysis and interpretations.

All authors contributed to the writing of the manuscript, read and approved the final version of the manuscript.
